# The Value of CT-Based Radiomics for Predicting Spread Through Air Spaces in Stage IA Lung Adenocarcinoma

**DOI:** 10.3389/fonc.2022.757389

**Published:** 2022-07-08

**Authors:** Xiaoyu Han, Jun Fan, Yuting Zheng, Chengyu Ding, Xiaohui Zhang, Kailu Zhang, Na Wang, Xi Jia, Yumin Li, Jia Liu, Jinlong Zheng, Heshui Shi

**Affiliations:** ^1^Department of Radiology, Union Hospital, Tongji Medical College, Huazhong University of Science and Technology, Wuhan, China; ^2^Hubei Province Key Laboratory of Molecular Imaging, Wuhan, China; ^3^Department of Pathology, Union Hospital, Tongji Medical College, Huazhong University of Science and Technology, Wuhan, China; ^4^Clinical Solution, Philips Healthcare, Shanghai, China

**Keywords:** adenocarcinoma, radiomics, spread through air spaces, lung cancer, stage IA

## Abstract

**Objectives:**

Spread through air spaces (STAS), a new invasive pattern in lung adenocarcinoma (LUAD), is a risk factor for poor outcome in early-stage LUAD. This study aimed to develop and validate a CT-based radiomics model for predicting STAS in stage IA LUAD.

**Methods:**

A total of 395 patients (169 STAS positive and 226 STAS negative cases, including 316 and 79 patients in the training and test sets, respectively) with stage IA LUAD before surgery were retrospectively included. On all CT images, tumor size, types of nodules (solid, mix ground-glass opacities [mGGO] and pure GGO [pGGO]), and GGO percentage were recorded. Region of interest (ROI) segmentation was performed semi-automatically, and 1,037 radiomics features were extracted from every segmented lesion. Intraclass correlation coefficients (ICCs), Pearson’s correlation analysis and least absolute shrinkage and selection operator (LASSO) penalized logistic regression were used to filter unstable (ICC < 0.75) and redundant features (r > 0.8). A temporary model was established by multivariable logistic regression (LR) analysis based on selected radiomics features. Then, seven radiomics features contributing the most were selected for establishing the radiomics model. We then built two predictive models (clinical-CT model and MixModel) based on clinical and CT features only, and the combination of clinical-CT and Rad-score, respectively. The performances of these three models were assessed.

**Results:**

The radiomics model achieved good performance with an area under of curve (AUC) of 0.812 in the training set, versus 0.850 in the test set. Furthermore, compared with the clinical-CT model, both radiomics model and MixModel showed higher AUC and better net benefit to patients in the training and test cohorts.

**Conclusion:**

The CT-based radiomics model showed satisfying diagnostic performance in early-stage LUAD for preoperatively predicting STAS, with superiority over the clinical-CT model.

## Introduction

In 2015, spread through air spaces (STAS), a novel invasive pattern in lung cancer, was recognized by the World Health Organization (WHO) Classification (1). STAS has been reported to occur in 14.8-56.4% of lung adenocarcinomas (LUADs) (2–5). Recent reports have shown that the presence of STAS is an independent risk factor for recurrence and low overall survival in small or early-stage LUAD (4, 5). Moreover, preoperative detection of STAS could help choose an appropriate surgery type (6, 7). Correspondingly, Ren et al (6) reported patients with STAS undergoing sublobar resection have a higher rate of pulmonary metastases than patients with STAS administered a lobectomy (25.8% vs 8.2%). Another study (7) showed the presence of STAS is associated with higher cumulative incidence of recurrence (CIR) and death (CID) in patients with sublobar resection compared with those undergoing lobectomy (5-year CIR, 39% vs. 16%; 5-year CID, 16% vs. 8%). Thus, it is critical to determine the STAS status in LUAD prior to the surgical decision-making.

It has been previously reported that some CT findings, including maximum tumor diameter, nodule type and percentage of the solid component, are related to STAS, with promising diagnostic efficacy (0.64-0.77) (8–11). However, the identification of these CT-based morphological features depends on the radiologist’s experience. Furthermore, the use of such qualitative CT features to predict STAS could inevitably lead to inestimable misdiagnosis and overdiagnosis. Radiomics is a characterization algorithm that can extract and analyze a large number of quantitative image features from medical images (12). Numerous studies have revealed the promising potential of radiomics in predicting gene mutations (13, 14), lymph node metastasis (15), therapeutic response (16) and clinical prognosis (17) in lung cancer. Two recent studies performing radiomics analysis of STAS have predicted the existence of STAS by establishing different models (18, 19); the established radiomics models achieved moderate performances for STAS prediction with areas under the curves (AUCs) of 0.63 and 0.754, respectively. However, none of the two studies compared the radiomics signature-based and clinical or CT morphological features-based models. This is of great interest because the introduction of radiomics into routine the clinical workflow is unlikely to be accepted if it does not provide additional predictive value compared to clinical factors or morphological CT features. Consequently, in the present study, in addition to establishing a radiomics model and assessing its capabilities, we simultaneously developed the clinical-CT and mixed models, and compared their predictive values for the STAS status.

## Materials and Methods

This retrospective study was approved by the Ethics Committee of Wuhan Union Hospital (S377), and the requirement for written informed consent was waived.

### Patients and Inclusion Criteria

**Figure 1:**
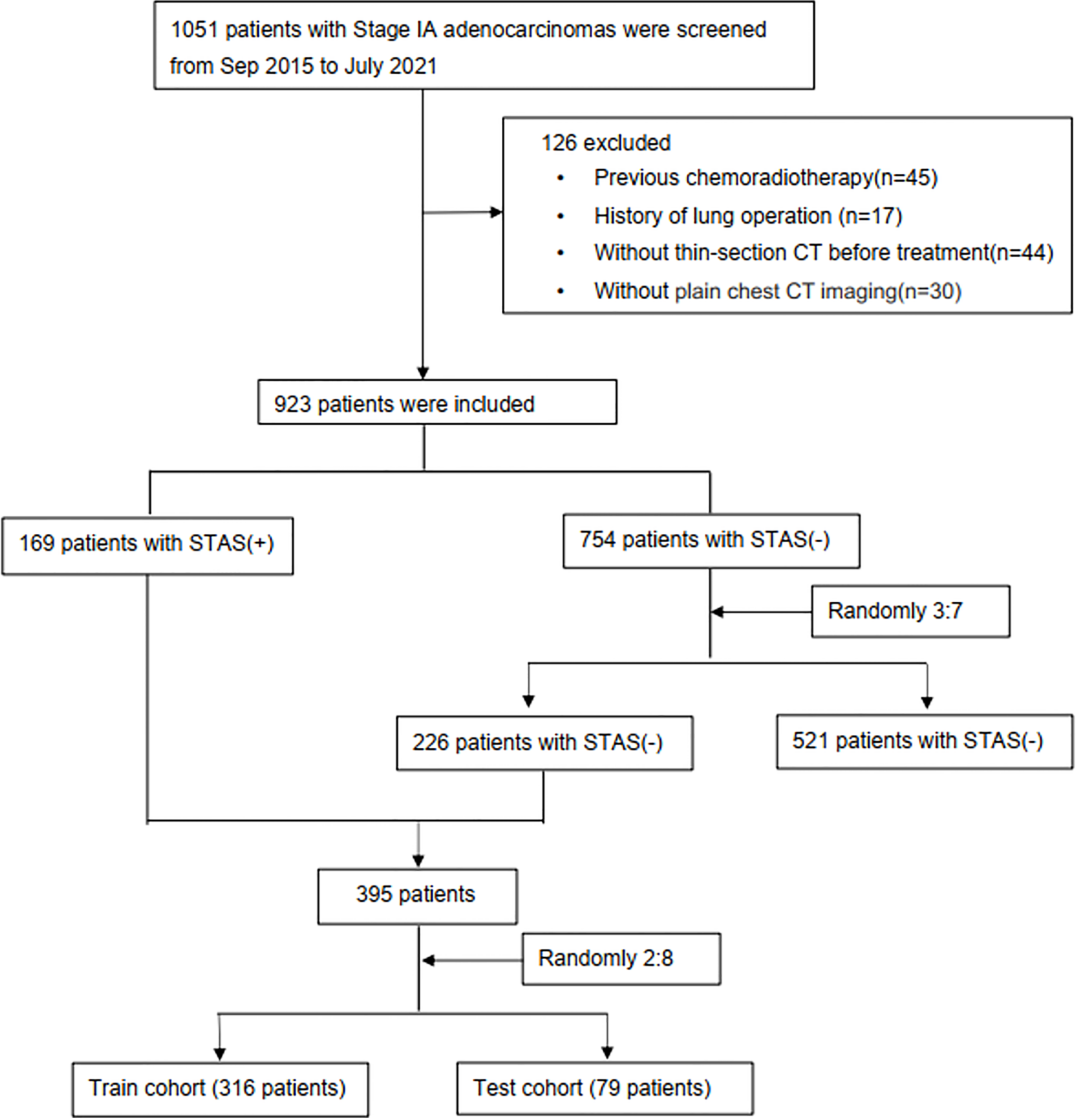
Study flowchart. STAS, spread through air spaces; STAS+, presence of STAS; STAS-, absence of STAS.

A total of 1051 patients with Stage IA adenocarcinoma (T1a-cN0M0) confirmed by curative surgery between September 2015 and July 2021 in Wuhan Union Hospital were retrospectively assessed. Then, 126 patients were excluded according to the following exclusion criteria (1): previous chemoradiotherapy (n=45); (2) a history of lung operation (n=17); (3) no thin-section CT before treatment (n=44); or (4) no plain chest CT imaging (n=30, **Figure 1**). As shown in **Figure 1**, the incidence of STAS in our institution was relatively low (169/923,18.3%). To overcome potential data imbalance, we randomly divided the STAS-negative cases by 3:7 into groups and matched them with the STAS-positive group at a nearly 1:1 ratio. Such method for balancing data has been validated in previous studies (11, 18, 20). Due to the fact that the incidence of STAS negative is higher than that of positive cases, we divided the negative data by a ratio of 3:7(226 cases) rather than 2:8(150 cases) to ensure the STAS negative cases were slightly larger than the positive ones. In addition, we had reperformed these models by 2:8 ratio for divide the negative data, and found that this ratio does not affect our main conclusions. In total, 395 patients (226 STAS negative and 169 STAS positive cases) were included, which were randomly assigned into training database (316 patients) and test dataset (79 patients), with a ratio of 0.8:0.2. In the train cohort, 136 patients were presented as STAS positive while 180 were negative. In the test cohort, 33 cases were positive for STAS while 46 were negative for STAS. All included patients had single lung adenocarcinoma.

The patients’ clinical characteristics and pathological findings, including age, sex, smoking history, histologic subtypes, surgical margin, lymphatic metastasis, epidermal growth factor receptor (EGFR) mutations and anaplastic large-cell lymphoma kinase (ALK) were recorded. A history of smoking was defined as lifetime exposure to more than 100 cigarettes. TNM staging was performed according to the IASLC 8th TNM Lung Cancer Staging System (21).

### Histopathological Analysis

Two pathologists (JF and NW, with 4 and 10 years of experience in thoracic pathology, respectively) blinded to clinical findings re-evaluated the hematoxylin and eosin-stained slides of all included patients using a multiheaded microscope. The predominant subtype of lung adenocarcinoma was assessed based on the International Association for the Study of Lung Cancer/American Thoracic Society/European Respiratory Society multidisciplinary classification of LUAD. STAS positivity was defined according to the WHO definition of STAS as tumor cells were found in the lung air spaces beyond the edge of the primary tumor, which is mainly composed of the following three forms: (1) air spaces filled by micropapillary structure without central fibrovascular cores; (2) air spaces filled by the solid component of the tumor; (3) air spaces filled by multiple discrete and single cells(1). After independent assessments, differences were resolved by consensus.

### CT Acquisition

CT was performed on two multislice spiral CT scanners (SOMATOM Definition AS+ and Siemens Healthineers, Germany) at our institution, using the following parameters: detector collimation widths, 64 x 0.6 mm and 128 x 0.6 mm; tube voltages, 120 kV. The tube current was regulated by an automatic exposure control system (CARE Dose 4D). Images were reconstructed at a slice thickness of 1.5 mm or 1 mm and an interval of 1.5 mm or 1 mm. No contrast medium was used. Digital Imaging and Communications in Medicine (DICOM) images from the picture archiving and communication system (PACS) were imported to the 3D-slicer software.

Two senior radiologists (HSS and JLZ, with 31 and 25 years of experience in thoracic radiology, respectively) evaluated the CT images to determine tumor size (longest diameter in MPR images), tumor density type (solid, mix ground-glass opacities [mGGO], and pure GGO [pGGO]), and GGO ratio (GGO diameter/tumor diameter) in consensus on the PACS. These specific CT features were chosen as the most contributing risk factors for STAS in patients with LUAD (8–11). The above two radiologists blinded to clinical and histologic findings assessed CT features on both axial CT and multiplanar reconstruction (MPR) images.

### Radiomics Feature Extraction

**Figure 2:**
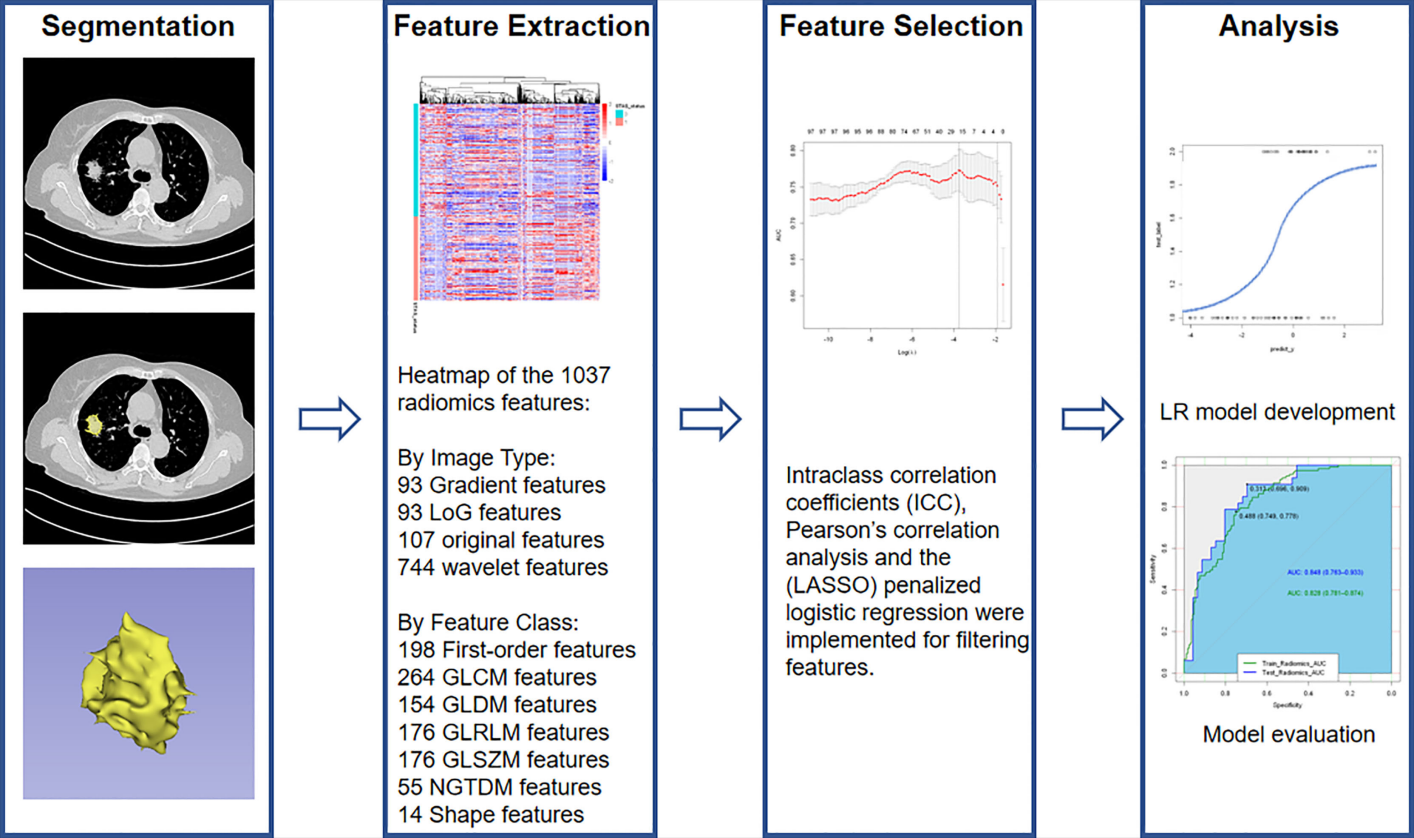
Radiomics workflow.

The regions of interest on CT images were semi-automatically delineated layer by layer by three junior radiologists (XH, YZ and JX with 5, 3 and 2 years of experience in thoracic imaging, respectively). All the three radiologists were aware of tumor presence and location but unaware of the pathological reports and STAS status. The 3D-slicer software was used for segmenting the lesions on each slice of CT scans semi-automatically and independently. Then, the three-dimensional volumes of interest (3D-VOIs) of tumors were automatically reconstructed with the 3D-slicer software (**Figure 2**). Two senior radiologists (HSS and JLZ) were responsible for checking all tumor segmentations, and any deviations were addressed with additional corrections. As reported in a recent publication (22), the semi-automatic segmentation from the 3D-Slicer is a better alternative to manual segmentation, as it can produce more robust and reproducible radiomic features. In addition, we randomly selected 40 cases at a ratio of 1:10 of all sample (396 cases) for estimating intraclass correlation coefficients (ICCs) analysis, which could ensure the precision and assurance of results (23). One observer (XH) repeated the segmentation after one week for intra-observer variability analysis. The other observer (YZ) performed the segmentation on the same image set using the same method, for inter-observer variability analysis.

The Philips Radiomics Tool (Philips Healthcare, China) was used for radiomics feature extraction, and core feature calculation was based on pyRadiomics (24). A B-spline curve interpolation algorithm was used to resample each 3D CT image to a spacing of (0.7, 0.7, 1.0) mm. For each VOI, a total of 1037 3D-radiomic features, including direct, wavelet transformed, logarithm transformed and gradient filtered features, were extracted (types and numbers are shown in **Figure 2**, and details can be also found at pyradiomics.readthedocs.io/en/latest/features.html).

### Statistical Analysis

The SPSS software (SPSS, version 21, IBM, Chicago, IL, USA) and R (version 4.0.2; http://www.Rproject.org) were used for all statistical analyses. LASSO binary logistic regression was performed with the ‘glmnet’ package. Multivariate binary logistic regression was carried out with the ‘rms’ package. Decision curve analysis was performed with the ‘rmda’ package. Receiver operating characteristic (ROC) analysis was carried out with the ‘pROC’ package. Descriptive analysis was performed to describe the distribution of the variables of interest for the training and testing cohorts. All continuous data and categorical variables were expressed as mean ± standard deviation and frequency (percentage), respectively. Independent samples Student’s *t* test was applied to compare continuous data in two groups, and the chi-square test was used to compare categorical variables. *P*<0.05 (two-tailed) was considered statistically significant.

All radiomics features were normalized to the z-score. Intraclass correlation coefficient (ICC) determination and Pearson’s correlation analysis were performed to exclude redundant and unstable features (r>0.8, ICC>0.75). ICC < 0.5, between 0.5 and 0.75, between 0.75 and 0.9, and > 0.90 indicated poor, moderate, good, and excellent reliability, respectively (25). Least absolute shrinkage and selection operator (LASSO) analysis was performed to determine features for further assessment (26). Five-fold cross-validation and maximum area under the curve (AUC) were used as feature filtering criteria. A classification model based on the selected features was established by a multivariate logistic regression (LR) algorithm. Feature selection was performed on the training set. In this work, we selected no model using the one-standard-error rule as the final model, because a five-fold cross-validation LASSO analysis was performed to filter features. A temporary model was built behind the LASSO analysis as to preserve features that are significant enough for the feature coefficients. More features kept after the LASSO analysis reflect a better performance for the final multivariate logistic regression algorithm (27). Three models were built based on three sets of features: a radiomics model based on the most contributing radiomics features from the temporary model, a clinical and CT model based on clinical and CT features only, and a mixed model (MixModel) combined the Rad-score with clinical and CT characteristics. The performances of these models for predicting STAS were evaluated in the training set, then in the test set by plotting ROC curves and calculating the areas under the curves (AUCs). The accuracy, sensitivity, specificity, negative predictive value (NPV) and positive predictive value (PPV) were then calculated for each model. The predictive ability was also illustrated by the confounder matrix. The DeLong test was conducted to compare diagnostic efficiency among the different models. Furthermore, decision curve analysis was preformed to determine the clinical usefulness of the three models by quantifying the net benefits at different threshold probabilities in the data set.

## Results

### Clinicopathological and CT Features of STAS

**Figure 3:**
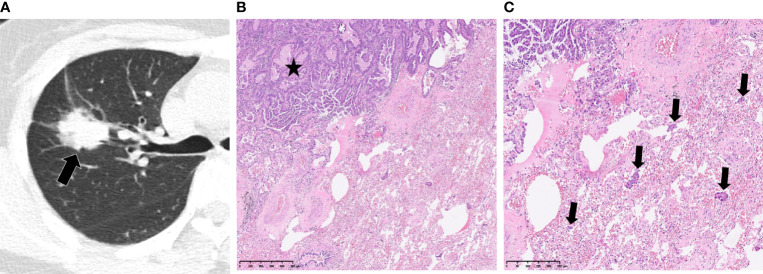
Spread through air spaces in a 52-year-old woman with papillary adenocarcinoma. **(A)** Axial CT image presenting a slightly lobulated, solid tumor in the right upper lobe (arrow). **(B, C)** Photomicrographs showing detached papillary clusters of tumor cells (arrows) in the alveolar space beyond the edge of the main tumor (*). Hematoxylin-eosin staining, magnification x50 **(B)**, x100 **(C)**.

**Figure 4:**
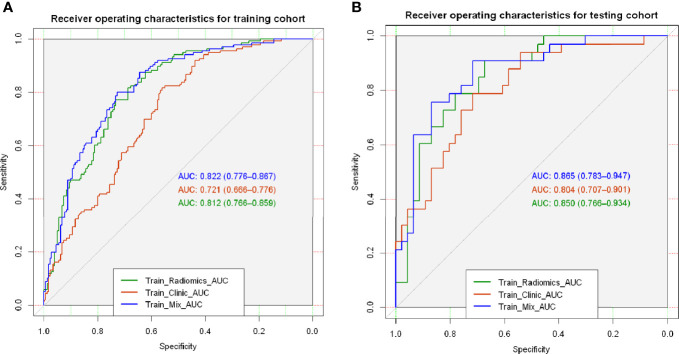
Performances of the three models in the training **(A)** and test **(B)** groups.

This study included 395 eligible patients (average age, 59 ± 10 years; 207 males) in total. The clinicopathological and main CT features of the final study population are shown in **Table 1**. STAS was found in 169/928 (18.2%) patients. No significant differences were found between STAS-positive and STAS-negative cases in age, gender and smoking history (*p*=0.268, *p*=0.232 and *p*=0.053, respectively). Concerning CT features, tumors with STAS tended to be larger than STAS negative counterparts (21 ± 6.3 mm vs 18 ± 6.7 mm, *p<*0.001). Tumor densities differed between the STAS and non-STAS groups (*p*<0.001). The majority of STAS-positive tumors manifested as solid nodules (152/169, 89.9%; **Figure 3**), followed by the mix GGO (13/169, 7.7%) and pure GGO (4/169, 2.4%) groups. Moreover, the less the GGO ratio, the higher the possibility of STAS positivity (0.08 ± 0.21 vs 0.38 ± 0.42, *p*<0.001). Then, age, gender, smoking history, diameter, density and GGO ratio were selected for predicting STAS in the clinical and CT model (Clinical-CT Model) by the LR algorithm (**Table 2**). Based on clinical and CT features, the model had an AUC of 0.721 (a sensitivity of 69.9% and a specificity of 61.7%) in the training cohort; the AUC was 0.804 (a sensitivity of 72.7% and a specificity of 76.1%) in the test cohort (**Figure 4**). The distribution of the selected clinical and CT features for the patients with STAS and those without STAS in the training and test cohorts are shown in **Table 3**.

**Table 1:** Associations of spread through air spaces with Clinicopathological features and CT findings.

Factor	Total patients	STAS (+)	STAS (-)	*p*-value
N	395	169	226	
Gender				0.268
Male	207 (52.4%)	94 (55.6%)	113 (50%)	
Female	188 (47.6%)	75 (44.4%)	113 (50%)	
Age, years	59 ± 10	60 ± 10	58 ± 10	0.232
History of smoking	63 (15.9%)	34 (20.1%)	29 (12.8%)	0.053
Diameter, mm	19 ± 6.7	21 ± 6.3	18 ± 6.7	<0.001*
GGO ratio	0.25 ± 0.37	0.08 ± 0.21	0.38 ± 0.42	<0.001*
Density				<0.001*
pGGO	39 (9.9%)	4 (2.4%)	35 (15.5%)	
mGGO	85 (21.5%)	13 (7.7%)	72 (31.9%)	
Solid	271 (68.6%)	152 (89.9%)	119 (52.7%)	
Histologic subtypes				<0.001*
Lepidic predominant	38 (9.6%)	4 (2.4%)	34 (15%)	
Acinar predominant	171 (43.3%)	74 (43.8%)	97 (42.9%)	
Micropapillary	26 (6.6%)	21 (12.4%)	5 (2.2%)	
Papillary predominant	106 (26.8%)	37 (21.9%)	69 (30.5%)	
Solid predominant	44 (11.1%)	30 (17.8%)	14 (6.2%)	
Mucinous predominant	10 (2.5%)	3 (1.8%)	7 (3.1%)	
Resection margin				0.013*
Negative	373 (94.4%)	154 (91.1%)	219 (96.9%)	
Positive	22 (5.6%)	15 (8.9%)	7 (3.1%)	
Pleural invasion				0.001*
Absence	358 (90.6%)	144 (85.2%)	214 (94.7%)	
Present	37( 9.4%)	25 (14.8%)	12 (5.3%)	
Perineural invasion				
Absence	379 (95.9%)	160 (94.7%)	219 (96.9%)	0.266
Present	16 (4.1%)	9 (5.3%)	7 (3.1%)	
EGFR				0.039*
Negative	154/261 (59%)	76/115 (59%)	78/146 (53.4%)	
Positive	107/261 (41%)	39/115 (33.9%)	68/146 (46.6%)	
ALK				
Negative	288/303 (95%)	11/124 (8.9%)	4/179 (2.2%)	0.009*
Positive	15/303 (5%)	113/124 (91.1%)	175/179 (97.8%)	

*P<0.05 based on comparisons between the two groups. Data are mean ± SD or n/N (%). STAS, spread through air spaces; STAS+, presence of spread through air spaces; STAS-, absence of spread through air spaces; EGFR, epidermal growth factor receptor; ALK, anaplastic large-cell lymphoma kinase; GGOs, ground-glass opacities; pGGO, pure GGO; mGGO, mix GGO.

**Table 2:** Features included in the clinical-CT model and their coefficients.

	Estimate	Std. Error	z value	Pr (>|z|)
(Intercept)	-1.10	1.25	-0.88	0.378
Sex	0.06	0.25	0.24	0.811
Gender	0.01	0.01	-0.18	0.861
Smoking	0.61	0.37	1.68	0.093
Size	0.03	0.02	1.70	0.089
GGO ratio	-1.59	0.99	-1.62	0.106
Solid nodule	0.65	0.99	0.66	0.509
mGGO	-0.25	0.71	-0.35	0.723

GGOs, ground-glass opacities; mGGO, mix GGO.

**Table 3:** Comparison of selected clinical and CT features between LUADs with STAS and those without STAS in the training and test cohorts.

	Train cohort			Test cohort		
	STAS(+) N=136	STAS(-) N=180	P value	STAS(+) N=33	STAS(-) N=46	P value
Gender	75 (55.1%)	92 (51.1%)	0.496	18 (54.4%)	20 (43.5%)	0.368
Age	59.4 ± 10.2	58.2 ± 10.3	0.310	60.0 ± 11.1	58.9 ± 9.1	0.660
Smoking	25 (18.4%)	22 (12.2%)	0.151	9 (27.3%)	7 (15.2%)	0.258
Size	21.3 ± 6.3	18 ± 6.7	<0.001	21.7 ± 5.9	18.2 ± 6.9	0.019*
GGO ratio	0.08 ± 0.20	0.38 ± 0.42	<0.001	0.08 ± 0.22	0.45 ± 0.40	<0.001*
Solid nodule	123 (55.6%)	100 (55.6%)	<0.001	29 (87.9%)	19 (41.3%)	<0.001*
mGGO	9 (6.6%)	49 (27.2%)	<0.001	4 (12.1%)	22 (47.8%)	0.001*

*P<0.05 based on comparisons between the two groups. Data are mean ± SD. STAS, spread through air spaces; STAS+, presence of spread through air spaces; STAS-, absence of spread through air spaces,GGOs, ground-glass opacities; mGGO, mix GGO.

Pathologically, the histologic subtypes differed between the STAS and non- STAS groups (*p*<0.001). Specifically, STAS-positive tumors had a lower frequency of lepidic predominant subtypes (2.4% vs 15%), while STAS-negative ones had a lower rate of solid predominant (6.2% vs 17.8%). The positive rate of resection margin was 5.6% (22/395) in this cohort and tended to occur more in STAS-positive tumors (8.9% vs 3.1%, *p*=0.013). Pleural invasion was observed more frequently in patients with tumors positive for STAS versus the STAS-negative group (14.8% vs 5.3%, *p*=0.001), whereas perineural invasion showed no significant difference (5.3% vs 3.1%, *p*=0.266). EGFR and ALK analyses were available in 261 and 303 patients, respectively. STAS positivity was associated with reduced incidence of EGFR (*p*=0.039) and higher incidence of ALK (*p*=0.009). Lymphatic metastasis was negative in all patients.

### Radiomics Model Building and Validation

**Figure 5:**
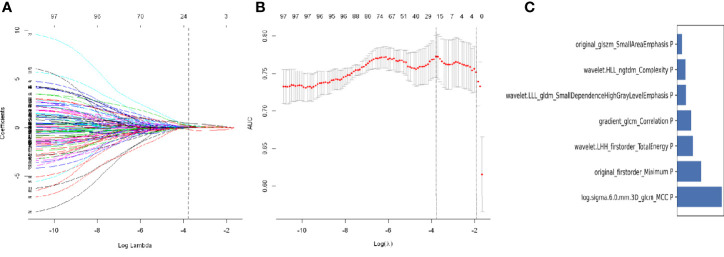
Least absolute shrinkage and selection operator (LASSO) logistic regression of radiomics features **(A)** and the regularization parameter λ **(B)**. **(C)** The feature weights of selected radiomics features.

After intraclass correlation coefficients (ICC) and Pearson’s correlation analysis, 98 radiomics features were selected for predicting STAS. Based on LASSO penalized logistic regression analysis, 18 features showed significant associations between radiomics and STAS (**Figures 5A, B**). Then, the top seven radiomic features with coefficients greater than 0.1 (two first-order and five second order parameters, including GLCM, GLSZM and GLDM features) were identified by the LR model (**Figure 5C**).

Finally, these seven radiomics features were entered into the LR model for building the radiomics model. Features contained in the radiomics model, and their coefficients are shown in **Table 4**. The radiomics model achieved good performance both in the training cohort(AUC: 0.812, sensitivity: 75%, and specificity: 78.3%) and the test cohort(AUC:0.850,; sensitivity:75.8%, and specificity:76.1%) (**Figure 4**).

**Table 4:** Features included in the radiomics model and their coefficients.

	Estimate	Std. Error	z value	Pr (>|z|)
(Intercept)	-0.76	0.20	-3.82	<0.001
wavelet.LHH_firstorder_TotalEnergy	0.49	0.20	2.50	0.012
wavelet.HLL_ngtdm_Complexity	0.21	0.186	1.15	0.249
wavelet.LLL_gldm_SmallDependenceHighGrayLevelEmphasis	0.46	0.28	1.68	0.093
log.sigma.6.0.mm.3D_glcm_MCC	1.28	0.52	2.46	0.014
gradient_glcm_Correlation	0.78	0.18	4.23	<0.001
original_glszm_SmallAreaEmphasis	0.30	0.23	1.30	0.194
original_firstorder_Minimum	0.77	0.20	3.73	<0.001

The rad-score of each lesion was calculated using the following formula:


$$\begin{array}{l} \text{Rad-Score}=0.585917936+0.49*\text{wavelet}.\text{LHH}\_\text{firstorder}\_\text{TotalEnergy}+0.21*\\ \text{wavelet}.\text{HLL}\_\text{ngtdm}\_\text{Complexity}+0.46*\\ \text{wavelet}.\text{LLL}\_\text{gldm}\_\text{SmallDependenceHighGrayLevelEmphasis}+1.28*\\ \text{log}.\text{sigma}.6.0.\text{mm}.3\text{D}\_\text{glcm}\_\text{MCC}+0.78*\text{gradient}\_\text{glcm}\_\text{Correlation}+\\ 0.30*\text{original}\_\text{glszm}\_\text{SmallAreaEmphasis}+0.77*\text{original}\_\text{firstorder}\_\text{Minimum} \end{array}$$

The rad scores for both the training and test sets are shown in **Figure S1**. STAS-positive tumors had significantly higher rad-scores than STAS-negative tumors in both the training and validation sets (p <0.001).

### Performance Comparison Among Different Models

**Figure 6:**
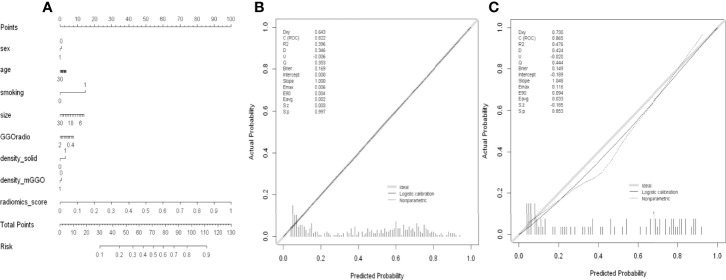
**(A)** Nomogram of MixModel for predicting presence of spread through air spaces(STAS). For each patient, draw a vertical line between the variable value and the corresponding point line, and then assign a score for each variable based on the clinical and imaging characteristics to obtain a total score. The risk of STAS can be predicted according to the total score. **(B)** Calibration curve for the MixModel in training cohort. **(C)** Calibration curve for the MixModel in validation cohort.

The MixModel of a comprehensive nomogram model was developed with the retain clinical CT characteristics(including age, gender, smoking history, diameter, density and GGO ratio) and rad-score(**Figure 6**), and showed AUCs of 0.822 and 0.865 in the training and test cohorts, respectively. Features contained in Mixmodel and their coefficients are listed in **Table 5**. Subsequently, we separately compared AUCs among MixModel, clinical-CT model and radiomics model (**Figure 4**). Both in the training cohort and the test cohort, the MixModel showed improvement in diagnostic ability compared with the clinical-CT model and radiomics model. In addition, the AUC of radiomics model was larger than that of the clinical-CT model in both cohorts (0.812 vs 0.721, *p*<0.001; 0.850 vs 0.804, *p*=0.228).

**Table 5:** Features included in Mixmodel and their coefficients.

	Estimate	Std. Error	z value	Pr (>|z|)
(Intercept)	-2.4	1.4	-1.8	0.08
Sex	-0.1	0.3	-0.2	0.8
Age	0.02	0.0	-0.1	0.9
Smoking	0.4	0.4	1.1	0.3
Size	0.0	0.0	-0.8	0.4
GGOradio	-0.4	1.1	-0.4	0.7
density_solid	0.4	1.1	0.4	0.7
density_mGGO	0.1	0.8	0.1	0.9
radiomics_score	5.1	0.7	7.1	0.0

GGOs, ground-glass opacities; mGGO, mix GGO

The results of confounder matrix analysis in the training and test sets in MixModel, and the clinical-CT and radiomics models are summarized in **Table 6**. The Mixmodel model showed the highest accuracy (number of correct predictions divided by that of total predictions) among the three models in the training set and in the test set.

**Table 6:** Confounder matrix for the training and testing sets in the three models.

Predicted results	Actual results		Accuracy (%)	Sensitivity (%)	Specificity (%)
	STAS (-)	STAS (+)			
**Clinical-CT model**					
Training data set			65.2	69.9	61.7
STAS(-)	111	41			
STAS(+)	69	95			
Testing data set			74.7	72.7	76.1
STAS(-)	35	9			
STAS(+)	11	24			
**Radiomics model**					
Training data set			76.9	75	78.3
STAS(-)	141	34			
STAS(+)	39	102			
Testing data set			76	75.8	76.1
STAS(-)	35	8			
STAS(+)	11	25			
**MixModel**					
Training data set			78.5	75	81.1
STAS(-)	146	34			
STAS(+)	34	102			
Testing data set			79.7	74.3	80.4
STAS(-)	37	7			
STAS(+)	9	26			

Rows correspond to the prediction of the logistic algorithm, and columns to known outcomes. STAS, spread through air spaces; STAS+, presence of spread through air spaces; STAS-, absence of spread through air spaces.

### Calibration Analysis and Clinical Use

For the radiomics model, calibration curve analysis showed P values of 0.954 and 0.792 in the training and test sets, respectively, indicating a good degree of fit for the model in both sets (**Figure S2**). Similarly, the clinical-CT model also showed good calibration abilities in both the training and test sets (**Figure S2**). The clinical usefulness of the three predictive models were examined by decision curve analysis (**Figure S3**). Compared with the treat-all and treat-none models, MixModel, and the clinical-CT and radiomics models could bring net benefits to patients, among which the radiomics model had the best benefit, while the clinical-CT model had the lowest.

## Discussion

In this study, we developed a CT-based radiomics model with good performance, which was superior to the clinical-CT model established by demographic characteristics and selected CT features, indicating the discrimination value of radiomics features for STAS positivity in IA-stage LUAD cases before surgery. Furthermore, MixModel also outperformed the clinical-CT model, presenting a higher value in predicting STAS. This report provides a powerful tool for preoperative decision-making in early-stage LUAD patients

As expected, this study showed that STAS-positive tumors tended to be larger, solid tumors with reduced GGO ratio on CT images, which was consistent with previous studies (8–11). In addition, Kim et al. (11) found that a predictive model using the percentage of the solid component could achieve an AUC of 0.77 for STAS detection. In this study, the clinical-CT model also had a similar AUC of 0.721 in the training cohort, and an AUC of 0.804 in the test cohort. The clues behind the associations of imaging features with STAS could be found in pathological findings. Several studies have revealed the connections between STAS and pathological characteristics (28, 29). Similar to previous studies (28), STAS-positive tumors in the present study had a high frequency of micropapillary, papillary or solid pattern growth, which might partially explain the association of STAS with solid tumor predominance on CT images. This study also found that STAS-positive tumors tended to be along positive resection margin and pleural invasion. In addition, the current study further analyzed the correlation between genetic mutations and STAS. We found that STAS positivity was associated with lower incidence of EGFR mutations and higher incidence of ALK mutations. Meanwhile, previous investigations have confirmed that the occurrence of GGOs is significantly associated with EGFR mutations (30) and the presence of solid nodules is one of the vital CT features of ALK rearrangement in LUAD (31). Thus, taken together, we might reasonably consider that STAS could be a potential factor in tumor aggressiveness. The larger size and solid nature on CT scans in this study supported such biological behavior.

In this study, the AUCs of the radiomics model were 0.812 and 0.850 in the training and test sets, respectively. Totally, seven features with coefficients >0.1 were selected for the tumors, including three first-order and seven second order indexes, including GLCM, GLSZM and GLDM features. First-order statistics are defined as the distribution of voxel intensity within the image region delineated by the mask through commonly used and basic metrics, while second order parameters involve the spatial position relationship with voxel intensity. Accordingly, many gray level features inferring intratumor heterogeneity were included in the radiomics model, suggesting that gray level features can contribute to the high diagnostic accuracy observed. Moreover, the present results demonstrated that the firstorder_Minimum feature was closely related to STAS, with the highest estimate coefficient (0.77) among the selected first-order parameters. The firstorder_Minimum feature referred to the lowest gray level intensity within the tumor, and STAS-positive tumors had higher firstorder_Minimum values than STAS-negative tumors. Therefore, these findings suggested that STAS-positive tumors tend to be more heterogeneous and solid components. Similarly, previous reports (18, 19) have also shown correlations between radiomics features representing gray level characteristics and STAS-positive tumors, such as Size-zone non-uniformity and Grey level variance. In addition, as shown in our clinical-CT model, the solid-density type and lower GGO ratio were the most critical features determining STAS risk. Since the automatic extraction of radiomics features by computer is more objective and accurate than subjective and manual measurements, our study confirms the reliability and interpretability of the features extracted by the radiomics analysis.

Two previous studies have explored the relationships between CT-based radiomics features and STAS in lung adenocarcinomas (18, 19). Chen et al. (19) developed a Naïve Bayes model using five radiomics features to predict STAS that achieved AUCs of 0.63 and 0.69 in the internal and external validation sets, respectively. Another report by Jiang et al. (18) built a random forest model using 12 CT-based radiomics features and showed a good AUC of 0.754 for predicting STAS. Our radiomics model established by LR outperformed those in the above two studies for STAS prediction (0.828,0.848). In addition to the differences in modeling methodologies, the discrepancy among the three studies might be related to patient inclusion criteria, sample size, and different data compositions. Chen et al. included both stage IA and IB adenocarcinomas in their study, while Jiang et al. analyzed LUAD patients with no TNM stage restriction. Meanwhile, only stage IA adenocarcinoma (T1a-cN0M0) patients were included in the present study. In addition, the number of STAS-positive patients in this investigation was twice those reported in the above two studies. In addition, STAS-positive tumors accounted for nearly 50% of all cases in this study, while the STAS-positive rates in the above two studies were less than 30%. Therefore, further studies with larger samples and better design are needed to confirm the present results.

There were several limitations in this research. Firstly, since this was a single-center retrospective study, the present radiomics model was not verified by external data. Thus, further multicenter studies are needed to confirm our results. Secondly, our study only included patients who underwent surgery for removing tumors, which might exclude cases with small tumors. However, tumor lesions larger than 3 cm were ruled out. Thirdly, since CT was performed on two different scanners, image acquisition protocols were slightly different, which might lead to some bias. Fourthly, we only included the specific CT features supported by previous reports (8–11), so the results might not represent the total CT morphological characteristics of tumors. However, the associations of other CT findings (such as satellite nodules) in LUAD with STAS remain controversial (32). Moreover, we calculated the mean and standard deviation in the training and testing sets, separately, but it might be better for clinical deployment to determine all model hyperparameters in the training set alone. Finally, since the patients were examined from 2015 to 2021, whose follow-up time was limited, we did not evaluate the effects of STAS on patient outcome.

In conclusion, the CT-based radiomics model showed a satisfying diagnostic performance for preoperatively predicting STAS, which can provide decision-making support for treatment planning in stage-IA LUAD. Besides, this radiomics model outperformed the clinical-CT model, indicating the additional value of radiomics features for predicting STAS positivity in LUAD. However, since this was a single-center retrospective study, these conclusions need to be confirmed in further prospective multicenter studies.

## Data Availability Statement

The raw data supporting the conclusions of this article will be made available by the authors, without undue reservation.

## Ethics Statement

The studies involving human participants were reviewed and approved by Ethics Committee of Wuhan Union Hospital. The ethics committee waived the requirement of written informed consent for participation. Written informed consent was obtained from the individual(s) for the publication of any potentially identifiable images or data included in this article.

## Author Contributions

The acquisition, data explanation, and manuscript draft were completed by XH and JF. XH, YZ and KZ were responsible for the analysis of CT images and the delineation of VOIs. CD and XZ were responsible for data analysis and interpretation. YL and JL acquired the clinical information. JF and NW performed the pathological analysis. HS and JZ designed the study and made multiple revisions to the manuscript. All authors contributed to the article and approved the submitted version.

## Funding

This study was supported by the National Natural Science Foundation of China (grant number: 82071921).

## Conflict of Interest

Authors, CD and XZ were employed by Philips Healthcare.

The remaining authors declare that the research was conducted in the absence of any commercial or financial relationships that could be construed as a potential conflict of interest.

## Publisher’s Note

All claims expressed in this article are solely those of the authors and do not necessarily represent those of their affiliated organizations, or those of the publisher, the editors and the reviewers. Any product that may be evaluated in this article, or claim that may be made by its manufacturer, is not guaranteed or endorsed by the publisher.

## Supplementary Material

Supplementary Figure 1 The Rad-score of each lesion in the training set **(A)** and test set **(B)**. STAS+, presence of STAS; STAS-, absence of STAS.

Supplementary Figure 2 Calibration curves for the clinical-CT model **(A, B)** and radiomics model **(C, D)** in the training and test sets, respectively.

Supplementary Figure 3 Decision curves for the three predictive models.
