# Supplementary figures and images for: The Value of CT-Based Radiomics for Predicting Spread Through Air Spaces in Stage IA Lung Adenocarcinoma
Source: Front Oncol. 2022 Jul 8;12:757389. doi: 10.3389/fonc.2022.757389 (PMC9307661; doi:10.3389/fonc.2022.757389)

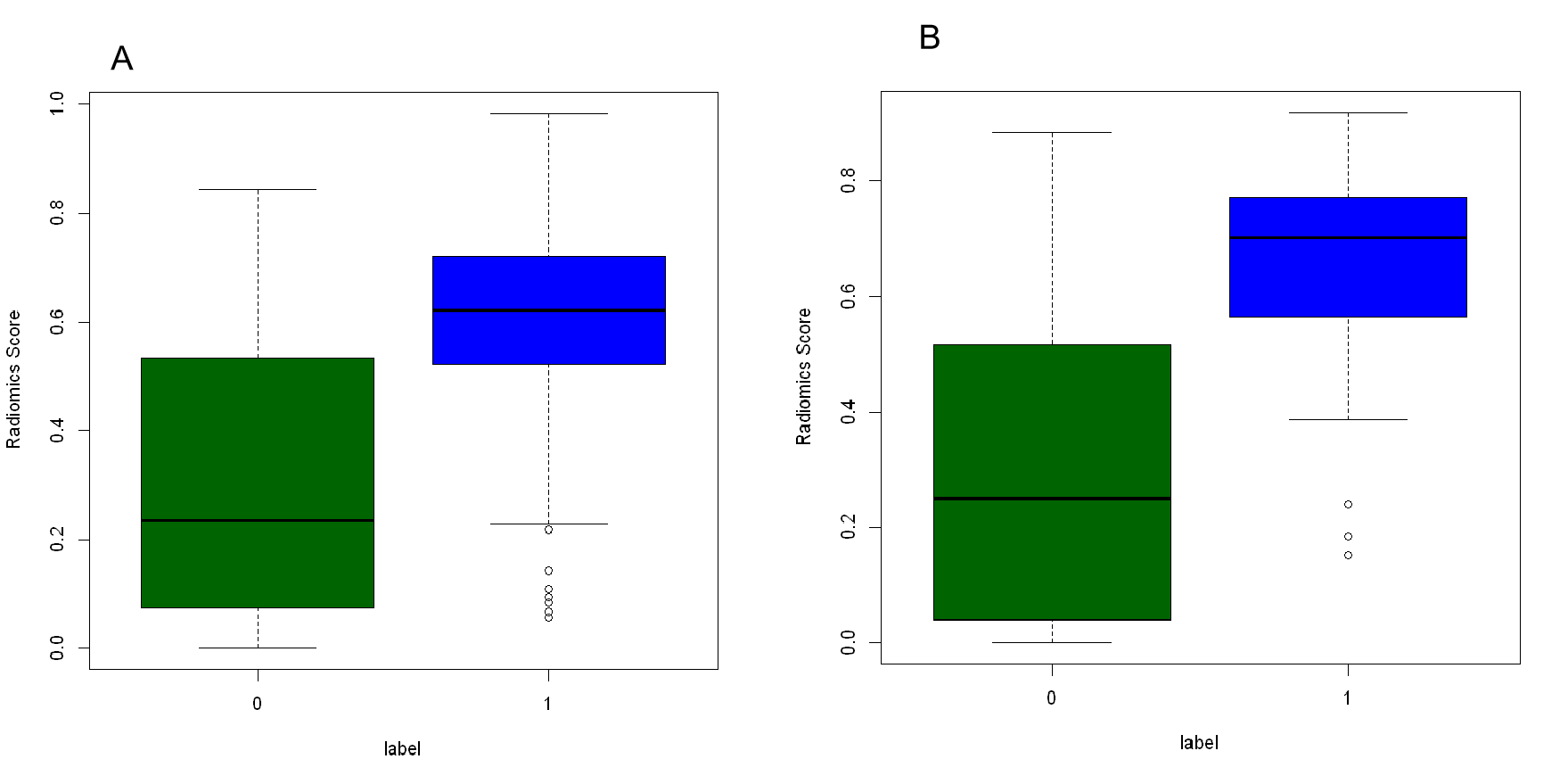

Supplement: Supplementary Figure 1 — The Rad-score of each lesion in the training set (A) and test set (B). STAS+, presence of STAS; STAS-, absence of STAS. [file Image_1.tif]

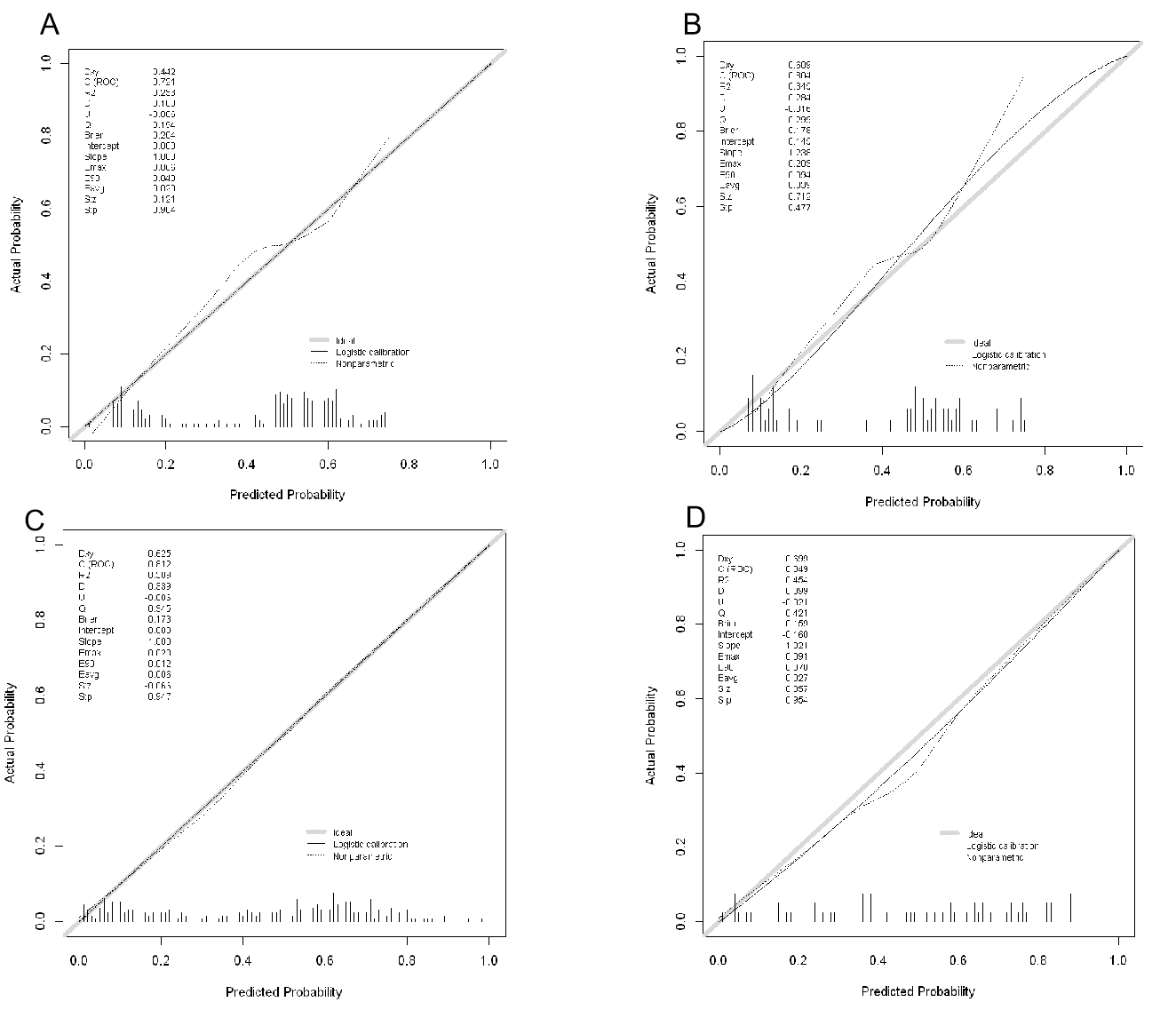

Supplement: Supplementary Figure 2 — Calibration curves for the clinical-CT model (A, B) and radiomics model (C, D) in the training and test sets, respectively. [file Image_2.tif]

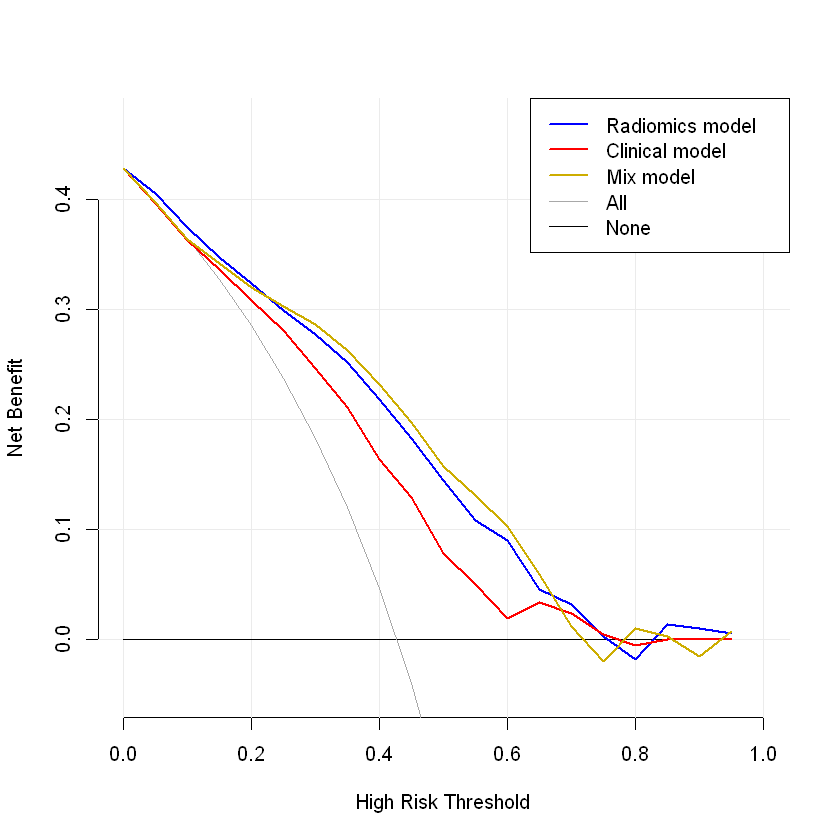

Supplement: Supplementary Figure 3 — Decision curves for the three predictive models. [file Image_3.tif]
